# Systemic inflammatory response syndrome in adult patients with nosocomial bloodstream infections due to enterococci

**DOI:** 10.1186/1471-2334-6-145

**Published:** 2006-09-26

**Authors:** Katharine Bar, Hilmar Wisplinghoff, Richard P Wenzel, Gonzalo ML Bearman, Michael B Edmond

**Affiliations:** 1 Department of Internal Medicine, Virginia Commonwealth University, Richmond, VA, USA; 2 Institute for Medical Microbiology, Immunology and Hygiene, University of Cologne, Cologne, Germany

## Abstract

**Background:**

Enterococci are the third leading cause of nosocomial bloodstream infection (BSI). Vancomycin resistant enterococci are common and provide treatment challenges; however questions remain about VRE's pathogenicity and its direct clinical impact. This study analyzed the inflammatory response of Enterococcal BSI, contrasting infections from vancomycin-resistant and vancomycin-susceptible isolates.

**Methods:**

We performed a historical cohort study on 50 adults with enterococcal BSI to evaluate the associated systemic inflammatory response syndrome (SIRS) and mortality. We examined SIRS scores 2 days prior through 14 days after the first positive blood culture. Vancomycin resistant (n = 17) and susceptible infections (n = 33) were compared. Variables significant in univariate analysis were entered into a logistic regression model to determine the affect on mortality.

**Results:**

60% of BSI were caused by *E. faecalis *and 34% by *E. faecium*. 34% of the isolates were vancomycin resistant. Mean APACHE II (A2) score on the day of BSI was 16. Appropriate antimicrobials were begun within 24 hours in 52%. Septic shock occurred in 62% and severe sepsis in an additional 18%. Incidence of organ failure was as follows: respiratory 42%, renal 48%, hematologic 44%, hepatic 26%. Crude mortality was 48%. Progression to septic shock was associated with death (OR 14.9, p < .001). There was no difference in A2 scores on days -2, -1 and 0 between the VRE and VSE groups. Maximal SIR (severe sepsis, septic shock or death) was seen on day 2 for VSE BSI vs. day 8 for VRE. No significant difference was noted in the incidence of organ failure, 7-day or overall mortality between the two groups. Univariate analysis revealed that AP2>18 at BSI onset, and respiratory, cardiovascular, renal, hematologic and hepatic failure were associated with death, but time to appropriate therapy >24 hours, age, and infection due to VRE were not. Multivariate analysis revealed that hematologic (OR 8.4, p = .025) and cardiovascular failure (OR 7.5, p = 032) independently predicted death.

**Conclusion:**

In patients with enterococcal BSI, (1) the incidence of septic shock and organ failure is high, (2) patients with VRE BSI are not more acutely ill prior to infection than those with VSE BSI, and (3) the development of hematologic or cardiovascular failure independently predicts death.

## Background

Over the past two decades, enterococcal bloodstream infections (BSI) have become increasingly pervasive. They account for a significant proportion of nosocomial BSI, and are now the third most common cause of nosocomial BSI [1]. Vancomycin resistance is common, found in approximately 2% of *E. faecalis *and 60% of *E. faecium *strains isolated in the United States [1]. Despite the increasing frequency of vancomycin-resistant enterococcal (VRE) infections, questions remain about VRE's pathogenicity and its direct clinical impact.

Patients with VRE BSI are often critically ill; it is difficult to determine whether the associated high mortality is directly attributable to the infecting organism or a marker of the patients' severe illness. Many studies have attempted to address this question, with conflicting results. Several have attributed high levels of morbidity and mortality to VRE [2-7]. Others have been unable to link increased mortality to vancomycin-resistance, demonstrating that vancomycin resistance is more of a marker of severe disease than a direct cause of poor outcome [8-11]. Most of the studies investigating enterococcal bloodstream infections (BSI), analyze predisposing factors and outcome measures, without closely examining the clinical course of patients through the infection. This study was conducted to evaluate the inflammatory response, clinical course and outcome of nosocomial bloodstream infections due to enterococci, as well as the effects of vancomycin resistance.

## Methods

### Setting

The Virginia Commonwealth University Medical Center (VCUMC) is an 820-bed, tertiary care facility in Richmond, Virginia. The hospital has nine intensive care units (ICUs), including pediatric ICUs and a burn unit. Approximately 30,000 patients are admitted annually. The study was approved by our Institutional Review Board.

### Study design

Using the Surveillance and Control of Pathogens of Epidemiological Importance (SCOPE) database [1], we identified all patients diagnosed with enterococcal BSI at VCUMC from November 2000 through December 2002. Patients were considered to have had an enterococcal BSI if they had at least one blood culture positive for this organism. Only monomicrobial BSI were included. Clinical data were retrospectively collected, including age, gender, location of the patient, clinical service, duration of hospitalization prior to onset of BSI, predisposing clinical conditions, the bloodstream pathogen and its antimicrobial susceptibilities. Predisposing clinical conditions included neutropenia (defined as an absolute neutrophil count of less than 500/μl), peritoneal or hemodialysis, mechanical ventilation, total parenteral nutrition, transfusion, antibiotic use, ICU stay, and intravascular catheters (i.e., central or peripheral intravenous catheters). Adverse outcomes that occurred during the hospital stay were recorded, including organ system failure and death. Accordingly, we evaluated only in-hospital mortality. Patients discharged from the hospital in less than seven days from the onset of BSI were assumed to be alive at seven days. The clinical condition of the patient was classified daily according to the systemic inflammatory response syndrome (SIRS) criteria (SIRS, sepsis, severe sepsis or septic shock) and APACHE II scores from two days prior through 14 days after onset of BSI. Patients who had nosocomial BSI due to vancomycin-resistant enterococci were compared with patients who had nosocomial BSI due to vancomycin-susceptible enterococci.

### Definitions

The date the positive blood culture was drawn was deemed Day 0 in the tracking of BSI. The patients' physiologic condition prior to the BSI and on the day of BSI were assessed using the APACHE II score. A cut-point of greater than 18 was used to stratify the severity of clinical condition. This methodology was used to be consistent with our previous studies in this area. The clinical condition of each patient during the bloodstream infection was classified daily as SIRS, sepsis, severe sepsis or septic shock using criteria previously published by the American College of Chest Physicians/Society of Critical Care Medicine (ACCP/SCCM). [12] Systemic inflammatory response syndrome (SIRS) was defined as two or more of the following: (1) temperature >38°C or <36°C, (2)heart rate >90 beats per minute, (3) respiratory rate >20 breaths per minute or a PaCO_2 _<32 mmHg, or (4) white blood cell count >12 × 10^9^/L or <4 × 10^9^/L or the presence of more than 10% immature neutrophils. Sepsis was defined as SIRS associated with *Enterococcus *isolated from at least one blood culture. Sepsis associated with organ dysfunction, hypotension or systemic manifestations of hypoperfusion constituted severe sepsis. Septic shock was defined as sepsis associated with hypotension unresponsive to intravenous fluid challenge or the requirement of a vasopressor agent. SIRS 0–4, severe sepsis, septic shock and death were used as mutually exclusive categories, with SIRS 0, 1, 2, 3, and 4 representing the sum of the clinical criteria used to score SIRS. The presence of organ system failure was assessed using the criteria described by Fagon [14]. Nosocomial infection and sources of infection were defined according to Centers for Disease Control and Prevention (CDC) criteria [14]. Adequate empiric antimicrobial therapy was defined as treatment administered within 24 hours of the positive blood culture with an agent to which the enterococcal isolate was susceptible. Per CDC definitions, if the patient had a central venous catheter present at the time of BSI and did not have an infection with the same organism at a different site, the infection was determined to be central venous catheter-related.

### Microbiological methods

Blood cultures were processed at the VCUMC clinical laboratory. After identifying to *Enterococcus *genus, a Strep API kit was used to determine species. Minimum inhibitory concentrations were determined using the E-test. Results were confirmed using a microbroth dilution method according to the Clinical and Laboratory Standards Institute. Vancomycin resistance was defined as an MIC ≥ 32 μg/mL.

### Statistical analysis

Results were expressed as a mean ± SD, or as a proportion of the total number of patients or isolates. For continuous variables, mean values were compared using two sample t-tests for independent samples. Differences in proportions were compared using a Chi-square test or Fisher's Exact Test, as appropriate. Mean values are reported ± SD. All tests of significance were two-tailed, with α set at 0.05. Independent predictors of the outcome of BSI were identified by means of stepwise logistic regression analysis, with a limit for entering and removing variables at 0.05. Our dependent variable was mortality, either 7-day or total in-hospital. All statistical analyses were done using SPSS software (SPSS Inc., Chicago, IL, USA).

## Results

### Study populations and patient characteristics

During the study period, a total of 330 nosocomial enterococcal BSIs were identified. Twenty-two were in pediatric patients (age <18 years), 245 were polymicrobial, and 13 had incomplete records. The remaining 50 monomicrobial BSIs caused by *Enterococcus *spp were analyzed (Table 1). Patients included in the study had a mean age of 52 years, and 50% of patients were female.

**Table 1:** Patient characteristics stratified by resistance pattern of infecting organism (VRE vs. VSE) and underlying severity of illness before infection.

	Total (n = 50)	VSE (n = 33)	VSE (n = 17)	AP≤18 (n = 38)	AP2>18 (n = 12)
Mean age (years)	52	53	49	48*	63*
Women	50%	48%	53%	55%	33%
Mean LOS prior to BSI (days)	21	16*	30*	22	18
Mechanical ventilation	60%	54%	71%	55%	75%
Hemodialysis	18%	12%	29%	13%	33%
TPN	12%	6%	23%	16%	0%
Transfusion	52%	48%	59%	50%	58%
Prior antibiotics	84%	78%	94%	86%	75%
ICU care	74%	73%	76%	76%	67%
Central venous catheter	88%	88%	88%	87%	92%
Neutropenia	6%	3%	12%	8%	0%
Vancomycin resistance	34%			34%	23%
AP2>18 at day 0	24%	24%	23%		
Mean time to appropriate antimicrobial therapy (days)	1.4	1.3	1.7	1.6*	0.8*

(APACHE II scores on day of onset of illness >18 or ≤ 18)

*P < 0.05

Forty-three (86%) patients had primary, catheter-related infections. Seven patients had secondary BSI, with four stemming from urinary tract infections and one each from an infected wound, an infected peritoneal fluid and an abscess. Thirty-seven (74%) of the nosocomial BSIs occurred in the ICU setting. The patients were nearly evenly divided among internal medicine and surgery services (48% were on the internal medicine service, 44% were on the surgery service, and 8% were on the hematology-oncology service). The admitting diagnoses were classified as hepatic (22%), cardiac (18%), cancer (12%), pulmonary (12%), GI (10%), trauma (10%), neurologic (8%) and transplantation (6%).

Among potential factors predisposing to BSI, intravascular devices were the most common, with 44 patients (88%) having central venous catheters. Prior to BSI, thirty patients (60%) required mechanical ventilation, while 26 patients (52%) received blood products, 9 patients (18%) underwent hemodialysis, 6 patients (12%) received total parenteral nutrition and 3 patients (6%) were neutropenic. The mean length of hospital stay prior to BSI was 21 days. Five patients (10%) were discharged within seven days of onset of BSI. Mean APACHE II score on day of BSI was 16.

### Microbiological features

Of the 50 enterococcal isolates, thirty (60%) were *E. faecalis *and seventeen (34%) were *E. faecium*. The remaining three isolates were E. durans, E. gallinarum and E. casseliflavus. Seventeen (34%) (all *E. faecium*) isolates were vancomycin resistant. Three of the vancomycin-susceptible isolates and all of the vancomycin resistant isolates were resistant to ampicillin. Length of hospitalization (mean) prior to BSI was longer for those patients with vancomycin-resistant than those with vancomycin-susceptible enterococcal BSIs (30 vs. 16 days, p < 0.05). The mean time to appropriate therapy was 1.4 days, without a statistically significant difference between the VRE and VSE groups.

### Clinical course

**Figure 1:**
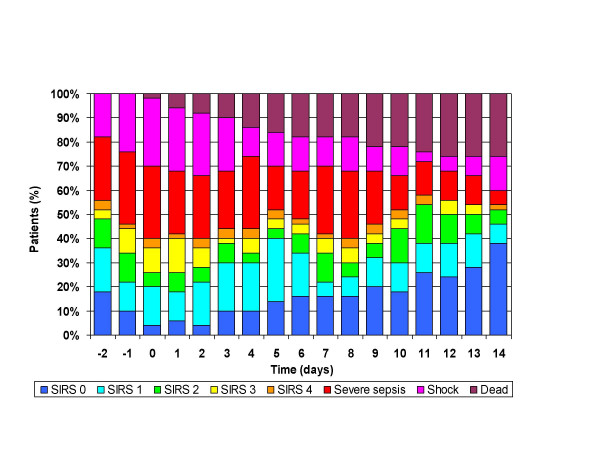
Systemic Inflammatory response (SIRS) over time in patients with enterococcal BSI.

Septic shock occurred in 62% of patients, and severe sepsis in an additional 18%, (figure 1). The incidence of organ failure was as follows: respiratory 42%, renal 48%, hematologic 44%, and hepatic 26%. Seven-day mortality was 18% and overall crude mortality was 48%, (table 2). Progression to septic shock was significantly associated with 7-day mortality (OR 8.5, p = 0.004) and overall mortality (OR 14.9, p < 0.001). The mean APACHE II score on the day of BSI was 16. The APACHE II score on the day 0 of the BSI was significantly associated with mortality (OR 8.0 for those with APACHE II>18, p = 0.005) (Table 2). There was no difference in APACHE II scores on days -2, -1, and 0 between the VRE and VSE groups. Maximal inflammatory response (MIR; proportion of patients with severe sepsis, septic shock or death) was seen on day 2 for VSE BSI vs. day 8 for VRE BSI and was significantly greater in those with VRE (Figure 3). No significant difference was noted in the incidence of organ failure, 7-day mortality or overall mortality between the two groups. Univariate analysis revealed that respiratory, cardiovascular, renal, hematologic and hepatic failure, as well as APACHE II score>18 two days prior to BSI were associated with death, but time to appropriate therapy >24 hours, age and infection due to VRE were not.

**Table 2:** Patient outcomes stratified by resistance pattern of infecting organism (VRE vs. VSE) and underlying severity of illness before infection.

	Total (n = 50)	VSE (n = 33)	VRE (n = 17)	AP2≤18	AP>18
Respiratory failure	42%	42%	41%	32%*	75%*
Cardiovascular failure	40%	42%	35%	34%	58%
Renal failure	48%	42%	59%	39%*	75%*
Hematologic failure	44%	33%*	65%*	42%	50%
Liver failure	26%	24%	29%	24%	33%
7-day mortality	18%	15%	23%	13%	33%
Overall mortality	48%	42%	59%	39%*	75%*

(APACHE II scores on day of onset of illness >18 or ≤ 18)

*P < 0.05

**Figure 2:**
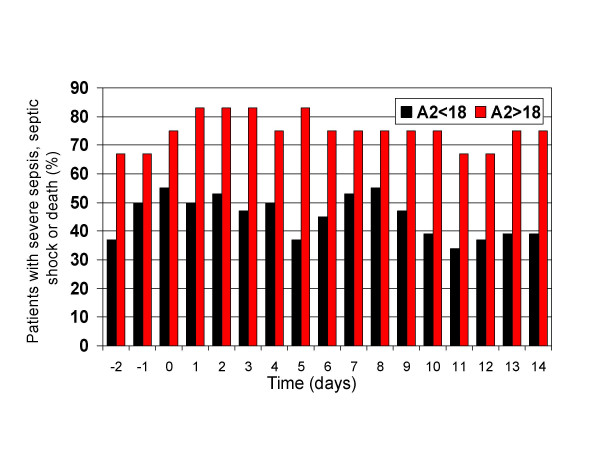
Severe sepsis, septic shock and death in patients with enterococcal BSI stratified by APACHE II score.

**Figure 3:**
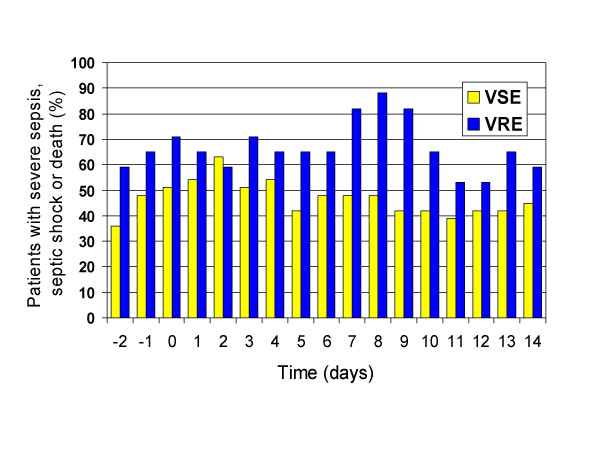
Severe sepsis, septic shock and death in patients with enterococcal BSI stratified by vancomycin resistance pattern.

Variables that were statistically significant in univariate analyses were selected for multiple logistic regression. Multivariate analysis revealed that hematologic failure (OR 8.6, p = 0.04) and cardiovascular failure (OR 7.6, p = 0.06) independently predicted death.

## Discussion

Our study investigated monomicrobial enterococcal BSI. Of the 308 infections in adult patients analyzed, only 16% (50) of these were monomicrobial, which is lower than in other reports [5,6]. The patients in our study were severely ill by several markers. The mean length of hospitalization prior to BSI was long (21 days), which is consistent with nationwide trends [1]. The majority (74%) were cared for in intensive care settings at the onset of their BSI. They also had significant mortality, with 18% 7-day mortality and 48% overall mortality. Our patients also had a high level of resistant infecting organisms; 34% of the enterococcal isolates were resistant to vancomycin. Many studies in the past 15 years have examined the role of vancomycin resistance on the outcome of enterococcal infections. Results have varied, with several demonstrating an association between vancomycin resistance and higher mortality. A 2003 meta-analysis of studies comparing outcomes in VRE and VSE bacteremia reported that, though most individual studies were insufficiently powered or adjusted to make this judgment, the available data suggested the VRE infections conferred higher mortality [7]. Few studies, however, have closely monitored patients' clinical courses throughout the hospital stay. We therefore investigated the systemic inflammatory response, clinical course and outcome of enterococcal BSI, stratifying by vancomycin susceptibility.

To characterize the severity of the patients' clinical condition and inflammatory response throughout the course of infection, we measured APACHE II scores and SIRS criteria from two days prior through 14 days after the onset of enterococcal BSI. Through univariate analysis, we found that patients' clinical state at onset of BSI influenced their mortality; patients with APACHE II>18 did significantly worse than those with APACHE II<18, (figure 2). When stratifying for vancomycin-susceptibility, there was no difference in APACHE II scores on the day of infection or the two days preceding infection between the VRE and VSE groups. Despite the similar clinical state at onset of infection, the VRE group had a greater inflammatory response that peaked later in hospitalization than the VSE group. Maximal inflammatory response was seen on day 2 for the VSE group and day 8 for the VRE group, (figure 3). Progression to septic shock was significantly associated with 7-day and overall mortality. The greater inflammatory response of VRE species, however, did not translate into different outcomes as there was no statistical difference in incidence of organ failure, 7-day mortality or overall mortality. Our study showed non-significant trends toward increased mortality in the VRE group, and it is possible that with the increased power of a larger sample size, the greater inflammatory response would have correlated with increased mortality.

The enterococcal isolates in our study were predominantly *E. faecalis *and *E. faecuim*. The vancomycin resistance pattern followed speciation, with all of the *E. faecalis *isolates being vancomycin susceptible and all of the *E. feacium *isolates being vancomycin resistant. This is somewhat divergent from national surveys which report 60% vancomycin-resistance in *E. faecium *isolates. [1] Additionally, this raises the possibility that other factors innate to the particular species, besides antimicrobial resistance pattern, could influence the clinical course in our analysis. Earlier investigations have associated *E. faecium *with higher mortality than *E. faecalis*; either because it tends to colonize and infect sicker patients or because it is inherently more virulent. [15] In a study controlling for species by comparing VRE vs. VSE in *E. faecium *species alone, no increase in mortality was associated with vancomycin resistance [9].

Similar to other studies, our analysis demonstrated longer hospitalization was a risk factor for VRE infection, with mean length of stay of 30 days in the VRE group and 16 days in the VSE group. Age, sex, prior antibiotic use, mechanical ventilation, hemodialysis, stay in intensive care setting, central venous catheter use and neutropenia were not significant risk factors for vancomycin resistance in this study.

Appropriate antimicrobial therapy within 24 hours of infection was not a significant determinant of mortality. This differs from past studies, where appropriate antimicrobials were more rapidly used in VSE infections. This likely reflects the evolution of available antimicrobials – with linezolid and quinupristin/dalfopristin being available during this study period, but not, or only on an investigational or compassionate-use-basis, in most earlier studies. These newer treatment options may change the mortality associated with VRE bacteremia. The more severely ill patients (those with APACHE II>18), did receive appropriate antimicrobial therapy significantly sooner (0.8 days versus 1.6 days), perhaps reflecting more aggressive clinical care or broader antibiotic coverage.

The presence of organ system failure was measured to further evaluate clinical course. Univariate analysis revealed that all measured forms of organ failure (respiratory, cardiovascular, renal, hematologic and liver) were associated with death. Multivariate analysis revealed that hematologic and cardiovascular failure independently predicted death. It is notable that hematologic failure, as defined by Fagon [13], includes the criterion of platelets <100,000/μL. This may have been affected by use of linezolid, which is associated with thrombocytopenia and may confound this result [16].

This study failed to associate an increased inflammatory response in VRE BSI to significantly increased mortality. The underlying clinical state was significant in determining outcome, whereas the resistance pattern of the infecting organism and appropriate antimicrobial therapy were not. This supports other studies in which the enterococcal BSI were found to have insignificant impact on clinical outcome. These results have been attributed to the low virulence of enterococcal species. However, similar results were found in several recent studies with common methodology analyzing more virulent pathogens (*Staphylococcus aureus*, *Candida *spp and *Pseudomonas aeruginosa*). In these analyses, the underlying clinical condition (as measured by APACHE II score) was also significant, while the resistance pattern or appropriate antimicrobial therapy were not. [17-19]. This furthers the concept that the host, rather than the pathogen, more greatly affects clinical outcome.

## Conclusion

The overall morbidity and mortality of patients with enterococcal BSI is high. Morbidity correlates closely with the clinical condition of patient at the time of infection as measured by APACHE II score, regardless of vancomycin susceptibility pattern. Though showing a trend toward greater and later peaking inflammatory state, vancomycin resistance did not predict outcome. Timely treatment with appropriate antimicrobials also did not predict outcome. However, hematologic and cardiovascular failure predicted death.

## Competing interests

The author(s) declare that they have no competing interests.

## Authors' contributions

KB conducted the data collection, participated in the statistical analysis and drafted the manuscript. MB conceived of the study, participated in statistical analysis and edited the manuscript. HW, RW and GB contributed to study design.

**Table 3:** Risk factors for mortality in patients with enterococcal BSI

	Univariate analysis		Multivariate analysis	
	OR	*P*	OR	*P*

Apache II score>18	4.6	0.03	3.2	0.28
Respiratory failure	8.0	0.005	3.6	0.15
Cardiovascular failure	9.7	0.002	7.5	0.03
Renal failure	3.9	0.049	0.8	0.88
Hematologic failure	6.4	0.01	8.4	0.02
Liver failure	5.9	0.015	3.3	0.26

## Pre-publication history

The pre-publication history for this paper can be accessed here:


